# The Synergistic Effects of Incobotulinum Toxin and Physiotherapy in a Rare Case of Paraparesis in a 7-Year-Old Affected by Klippel–Feil Syndrome Related to an *MYH3* Gene Mutation: A Case Report

**DOI:** 10.3390/jpm14111073

**Published:** 2024-10-24

**Authors:** Maurizio Ranieri, Mariagrazia Riccardi, Maria Vittoria Raele, Giacomo Farì, Marisa Megna, Riccardo Marvulli

**Affiliations:** 1 Department of Translational Biomedicine and Neuroscience (DiBraiN), Aldo Moro University, G. Cesare Place 11, 70125 Bari, Italy; maurizio.ranieri@uniba.it (M.R.); mariagrazia.riccardi7@gmail.com (M.R.); maryvi.92@hotmail.it (M.V.R.); marisa.megna@uniba.it (M.M.); 2 Department of Biological and Environmental Science and Technologies (Di.S.Te.B.A.), University of Salento, 73100 Lecce, Italy; giacomo.fari@unisalento.it

**Keywords:** Klippel–Feil syndrome, myosin heavy chain 3 mutation, *MYH3* mutation, spine defects, spasticity, botulinum toxin type A, incobotulinum, therapeutic exercise, physiotherapy

## Abstract

Background: Klippel–Feil disease is a condition characterized by a defect in the spine, consisting of the fusion or non-separation of two or more vertebrae of the cervical tract. It affects 1 in every 50,000 newborns, and the pathogenesis remains unknown to date, although the role of certain genes that are involved in segmentation processes is being studied. A single case of a genetic Myosin Heavy Chain 3 (*MYH3*) mutation is described here. Affected patients are typically distinguished by a relatively short neck, which leads to limited mobility, a low hairline, and obesity; they may also experience various other health issues. The common occurrence of comorbidities further diminishes the quality of life of these young individuals. Methods: The following case report describes the synergistic effect of Incobotulinum toxin type A and physiotherapy in a 7-year-old patient with MYH3 mutation-related Klippel–Feil syndrome (KFS) complicated by bilateral paraplegia to improve the spasticity condition of the lower limbs. To assess improvements over time, the patient underwent rating scales to determine spasticity (Modified Ashworth Scale: MAS), the neck’s range of motion (ROM), and muscle tone by using MyotonPro^®^. Specifically, measurements were taken on the day of the first medical examination (T0), the month after the injection and the startup of therapeutic exercise (T1), at three months (T2), and then once a month for a total of 6 months (T3, T4, and T5). Results: This therapeutic approach resulted in highly satisfactory outcomes for the child’s well-being, which was maintained until the sixth month and was accompanied by a complete absence of any side effects.

## 1. Introduction

Klippel–Feil disease is a condition characterized by a defect in the spine, consisting in the fusion or non-separation of two or more vertebrae of the cervical tract, which generally occurs between the third and the eighth gestation week [1]. Although the incidence is not clarified with certainty, one study defines it as about 0.2 cases per 1000 people [2]. The prevalence is about 1 in every 50,000 new infants worldwide, mainly affecting the female sex (60%) [3]. Most cases are sporadic, often incidentally diagnosed through radiographs taken for unrelated reasons [4]. Typically, patients present with a very short and minimally mobile neck due to vertebral fusion, a noticeably low hairline, and obesity. Additionally, these physical characteristics can significantly impair the quality of life for these young patients, as they might be associated with various malformations and other medical conditions. Among the most commonly reported in the literature are scoliosis, which occurs in approximately 60% of cases, and spina bifida occulta, seen in about 45%. Furthermore, there are also instances of concomitant cardiological, nephrological, and, unfortunately, neurological disorders [5].

It is readily apparent that the most significant challenges are associated with neurological disorders, which can vary widely from seizures to paraplegia. Current estimates suggest that approximately 15–20% of patients with KFS experience neurological complications. Therefore, addressing and managing these symptoms should be a priority for healthcare professionals and researchers, as it can greatly enhance the quality of life for children, allowing them to lead lives closer to those of their healthy peers. From the numerous studies reported in the literature, it seems that the major cause of these neurological issues is due to abnormalities of the occipitocervical region and spinal cord compression, which may occur both intrinsically, such as in canal stenosis, and extrinsically [6,7]. For instance, there is a documented case of a KFS patient who developed bilateral paraplegia as a result of a cervico-dorsal neuroenteric cyst [8].

Despite extensive research on KFS, its pathogenesis remains unclear to date. From what is known, it appears to be a congenital condition related to specific mutations in genes that are responsible for bone formation, development, and segmentation [9]. Among the key genes identified are growth differentiation factors 6 (GDF6) and 3 (GDF3), mesenchyme homeobox 1 (MEOX1), and, most notably, the myosin heavy chain 3 (*MYH3*) gene, which is crucial for the formation of the sarcomeric unit involved in the contractile apparatus of skeletal and cardiac muscle [10]. While monoallelic variants of this gene can lead to distal arthrogryposis [11,12,13], heterozygous variants can be a cause of contractures, pterygia, and spondylocarpotarsal fusion syndrome, a condition characterized by contractures and fusion of the vertebrae of the spine and carpal and tarsal bones [14]. Ultimately, it appears that the pathogenetic mechanism linked to variants of the *MYH3* gene consists of the inhibition of TGF-β signaling, which is critical to the osteoblastic and bone differentiation process [15].

Even though KFS turns out to be asymptomatic in most cases, it can prove to be a major problem for young patients with neurological complications. Thus, our specific study treats the case of a young girl with KFS associated with *MYH3* gene abnormalities with an accompanying major spastic paraparesis. The aim, therefore, is to demonstrate how, even in such a complex syndrome, synergistic treatment with Incobotulinum toxin and physiotherapy can be very helpful in the objective improvement of the patient’s lifestyle.

## 2. Case Report

**Figure 1:**
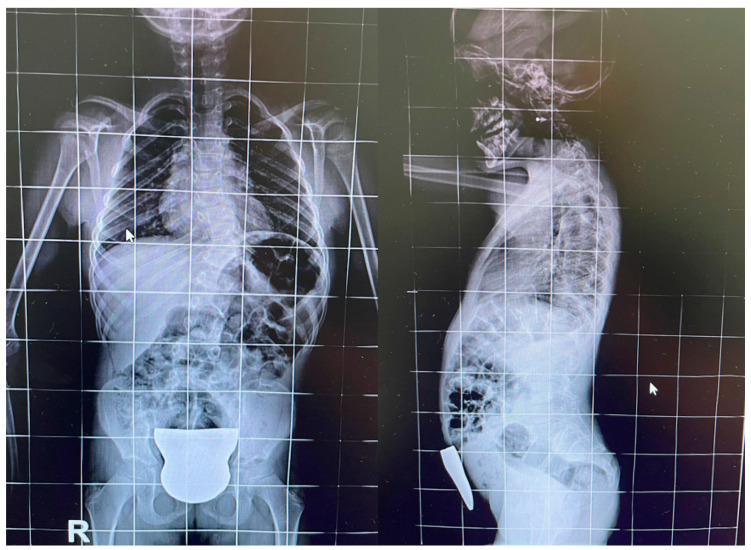
Antero-posterior and lateral X-rays of the patient’s spine.

**Figure 2:**
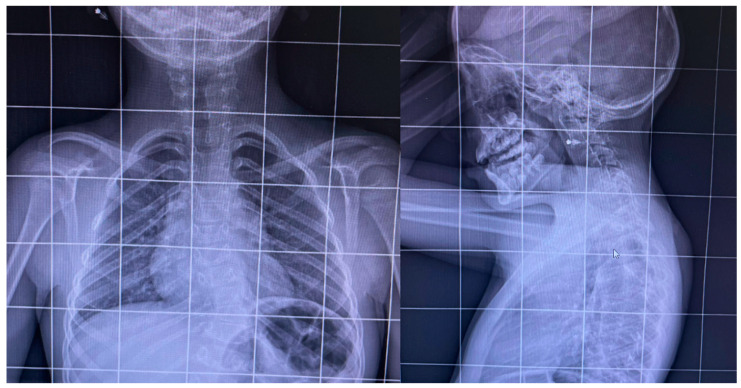
Zoomed-in antero-posterior and lateral X-rays of the patient’s spine.

A young patient, born in 2016 and diagnosed with KFS due to *MYH3* gene mutation and paraparesis (ASIA D with Medical Research Council less than or equal to 3 in principal muscles) at the age of three (2019), presented to the Bari “General Hospital”. The patient is a firstborn child and delivered at full term through spontaneous birth following a physiologically normal pregnancy. Early psychomotor development milestones were reported to be within the normal range. The parents reported that the onset of walking occurred at 14 months with anserine characteristics in the initial phase. In 2019, in response to the rigidity of the passive mobilization of the lower limbs, the girl was subjected to evaluation at the pediatric Metabolic Diseases and Genetics Unit at “Giovanni XXIII” Hospital, Bari, where, after careful analysis, hyposomy; left convex dorso-lumbar syndromic scoliosis derived from the posterior fusion of the dorsal vertebrae 9, 10, and 11 (D9-D10-D11) arches; fusion of the first two cervical vertebrae, atlas and the epistrofheus; and probable posterior fusion of the arches of D4-D5 and D2-D3 were observed (Figure 1 and Figure 2).

On this occasion, the little girl was diagnosed with a rare case of KFS, associated with paraparesis. Paraparesis is a condition in which there is a partial loss of the motor capacity of the limbs, most frequently the lower limbs. It differs from paraplegia where, on the other hand, there is a total loss of movement [16]. At the genetic analysis, a genomic variant c.800-1G>A in conditions of heterozygosis of the *MYH3* gene was found. The segregation analysis revealed the paternal origin of the variant. The patient underwent orthopedic evaluations for the management of scoliosis, for which surgical indication was excluded, and was followed by the neuropsychiatry and territorial rehabilitation service and by the endocrinology service for the treatment of hyposomia (currently treated with growth hormone, GH). Due to the finding of a knee flexion attitude, with functional limitation of walking and, consequently, recreational abilities, therapeutic exercise consisting of passive and active mobilization and muscular stretching of the lower limbs was started, without any benefit. Since pediatric treatment with antispasmodic drugs is not recommended and there was no indication to undertake intrathecal baclofen therapy, the patient was referred to our clinic for a careful evaluation. Considering that injection treatment with botulinum toxin type A and, specifically, Incobotulinum (Xeomin^®^, Merz Pharma, 60318 Frankfurt am Main Germany), was indicated, the patient was appropriately treated and discharged with an indication for physiotherapy (passive and active mobilization and muscular stretching of the lower limbs) to begin immediately. During the first medical examination, we collected the patient’s weight (20 kg), height (110.9 cm), sitting stature (53.9 cm), head circumference (48.8 cm), and lower limb length (58.1 cm), with the aim of making the study reproducible and demonstrating the effectiveness of the infiltrated toxin units in proportion to the age and all the data. The patient was evaluated before the combined therapy of BTX-A and therapeutic exercise (T0) and in subsequent follow-ups at, respectively, 30 days (T1), 90 days (T2), 120 days (T3), 150 days (T4), and 180 (T5) days after the beginning of the treatment, for a total of 6 months of observation.

We considered three parameters as benchmarks to study the progress of improvements over time, specifically the range of motion (ROM), which enables us to quantify the passive and active joint mobilization in degrees; the Modified Ashworth Scale (MAS) to study spasticity and muscular tone; and Biceps Femoris (BF) dynamic stiffness, evaluated using MyotonPRO^®^ (Myoton AS, Tallinn 11415, Estonia), which we have already used in other studies with very precise results [17,18,19,20].

At T0, a neurological visit showed normal mental status, speech, cranial nerve examination, and no sign of meningeal irritation. The physical examination showed that walking was possible for medium-long distances with bilateral slight flexion of the knees at 20° and hyporetropedal support (left > right). Resistance to passive mobilization was noticed, starting from the medium degrees in knee extension with a minus of 15° on the left and 10° on the right, as well as bilateral hypertonicity of the BF muscle (MAS = 3). The hip ROM was in the normal range bilaterally, probably due to a greater involvement of the short head of the affected muscle. Regarding the ankle joint, a slight limitation of the joint’s ROM at the extreme degrees in passive movement was noticeable but not so much as to require intervention to improve the gait pattern. In fact, the patient was not recommended a brace either. Achilleus Clonus could not be summoned. We registered absent hyperextension of the big toe and claws of the fingers. During the first evaluation, after collecting the written informed consent from the parents, we treated the girl’s BF with incobotulinum toxin type A (Xeomin^®^, Merz Pharma, 60318 Frankfurt am Main Germany), diluted in 1 cc of physiological solution, with 7 Units (U) for the left BF and 5U for the right BF via ultrasound-guided injection. Three days after the botulinum toxin injection, the patient undertook a specific physiotherapy program with the aim of promoting the diffusion of the toxin and enhancing its effects, with a frequency of two times a week.

## 3. Results

One month after the infiltrative and physiokinesitherapy treatment (T1), the patient was re-evaluated. During walking, the knee flexion showed persistent retropodal hyposupport on the left. In the supine position, the left knee was at 15°, while the right one was flexed at 10°, both of which were reducible to passive mobilization without any bilateral deficits. Three months later (T2), a further assessment was conducted. During walking, there was a noticeable knee flexion posture with continued retropodal hyposupport on the left side. In the supine position, the right knee was flexed 5° and completely reducible to passive mobilization, while the left knee was flexed at 10° and was completely reducible to passive mobilization. Given the sustained benefit from the infiltration, additional treatment with BTX-A was postponed, and a follow-up was scheduled for 1 month later (T3). At both T3 and T4, clinical and myometric improvements persisted and, therefore, the second infiltration was not performed. However, by T5, the clinical and myometric data indicated no further improvements, prompting the administration of a new infiltrative treatment (7U on the left and 5U on the right). The results are summarized in Table 1, Table 2 and Table 3.

The patient is currently being followed by our clinic, and no side effects have been reported.

## 4. Discussion

Botulinum toxin (BoNT) is a potent neurotoxin produced by Gram-positive spore-forming bacteria: *Clostridium botulinum* [21]. Its use is well established for conditions such as dystonia, spasticity, and sialorrhea, but its application is also spreading in the esthetic field [22]. Seven types of BoNTs are reported in the literature, differing from each other in their serological typing [23]. Among these, several subtypes have been identified, which differ in certain amino acid sequences and, as a result, have distinct toxo-pharmacological properties, denoted by an alpha numeric code [24]. In the medical field, commonly used products include *onaBoNT*, *aboBoNT*, and *incoBoNT*, all of which are serotype A, and *rimaBoN*, which is serotype B [25]. Typically, the toxin is available in vacuum-dried or lyophilized form, necessitating dilution with saline before use. After that, the resulting solution can be injected into muscles or specific sites, such as the salivary glands, a technique that is widely used in Parkinsonian patients suffering from sialorrhea. The mechanism of action of BoNT consists of the inhibition of the release of Acetylcholine (Ach) from the motor terminals and, thus, the inhibition of skeletal muscle contractions, even when the action potential exceeds a certain threshold [24]. Effects are usually observed within the first few weeks, peaking around thirty days post-injection. Although rare, instances of the product being ineffective may arise from the formation of antibodies against the toxin. Additionally, it seems that human serum albumin (HSA), which is normally used to stabilize the toxin, might induce the destruction of the toxin itself [26]. According to the US Food and Drugs Administration (FDA), among the listed toxins, *incoBoNT* is the one with the lowest concentration of HSA [26].

In treating spasticity, the first approach is to administer oral antispasmodic drugs, but resistance is unfortunately not very uncommon. Moreover, in addition to the classic problem of resistance, for young patients with KFS, taking pills is difficult to manage. Our case turns out to be unique.

As previously pointed out, the association of KFS with neurological symptoms is not particularly common: only 15–20% of affected patients may have concomitant mild disorders, such as paresthesia or synkinesis, but also more important and disabling pathologies, such as frequent seizures or gait alterations with ataxic walking or even para, hemi, or quadriplegia. Neurological sequelae can also depend on bone abnormalities. Specifically, all movements involving the neck should always be well controlled, if not completely avoided. In fact, as mentioned above, one of the main characteristics of patients with this syndrome is that they have limited neck mobility. In this sense, the movements to be avoided are not specific movements, but rather, it is necessary to conduct them slowly, whether in the direction of flexion and extension or rotation to the right or to the left. In this specific case, it is also a good idea to perform physiotherapy treatments exclusively with experienced personnel, as traumatic injuries even of a minor nature could prove to be very disabling. It is important to know that not all physical therapists are familiar with this rare condition, and as a result, some may think that forcing a little bit more every day will help in the recovery of movement, when, in fact, such behavior might just lead to complex issues. A study conducted in 1987 reports the occurrence of signs of spinal cord compression and quadriplegia after low-intensity trauma [27]. It appears, moreover, that a predisposition to easier spinal cord injuries is inherent to the disease. Indeed, vertebral fusion seems to lead to a certain hypermobility of the spine immediately above and below segments. In addition to the direct possibility of spinal cord damage given precisely by this hypermobility itself, there is also the possibility of indirect damage, with the formation of osteophytes over time, which can lead to major neurological sequelae even with minor trauma [28]. In addition, nerve root compression can lead to upper and lower neuron injury, resulting in spasticity, hyperreflexia, and ataxia [29]. Treatment is usually symptomatic, and surgical intervention tends to occur only in very rare cases with disabling symptoms that do not regress with specific medical therapy.

Since our patient was not experiencing any problems at the level of the cord and had not suffered any kind of trauma, we hypothesized that the associated paraparesis might somehow result from the very rare mutation found in the patient, of which we found no further cases in the literature. As already pointed out, the patient had no issues that could in any way hinder her from having a lifestyle that is as similar as possible to that of a person of her own age, except paraparesis. Therefore, we considered botulinum toxin treatment, hoping it could leave the patient free of this symptom for as long as possible, and enhanced the effect and duration by associating it with specific therapeutic exercise.

## 5. Conclusions

KFS is frequently an asymptomatic condition; however, it can be linked to a variety of anomalies. The most debilitating of these are often neurological issues, which, although rare, can affect the quality of life and normal development of the patients involved. In cases with additional manifestations, treatment is typically symptomatic, whereas in asymptomatic patients, the emphasis should be on avoiding sudden neck movements to prevent spinal cord injury. Specifically, our case report deals with a unique and rare instance of KFS in a child with a mutated *MYH3* gene and neurological manifestations such as paraparesis. This case illustrates how a combined treatment approach of therapeutic exercise and BTX-A injection into the BF muscles can provide benefits in the absence of side effects, resulting in a child with a lifestyle that is as similar as possible to that of her peers.

## Figures and Tables

**Table 1:** ROM assessment of right and left knee during the study; the data demonstrate improvement until 6 months after injection. () indicates minus.

	T0	T1 (30 Days)	T2 (90 Days)	T3 (120 Days)	T4 (150 Days)	T5 (180 Days)
**Right Knee**	20° (10°)	15° (0°)	5° (0°)	5° (0°)	7° (0°)	10 (5°)
**Left Knee**	20° (15°)	10° (0°)	10° (0°)	10° (0°)	10° (0°)	15° (7°)

**Table 2:** MAS assessment of right and left Biceps Femoris during the study; the data demonstrate improvement until 6 months after injection.

	T0	T1 (30 Days)	T2 (90 Days)	T3 (120 Days)	T4 (150 Days)	T5 (180 Days)
**Right Biceps Femoris**	3	1+	1+	1+	1+	3
**Left Biceps Femoris**	3	1+	1+	1+	2	3

**Table 3:** Myometric tone assessment of right and left Biceps Femoris during the study; the data demonstrate improvement until 6 months after injection.

	T0	T1 (30 Days)	T2 (90 Days)	T3 (120 Days)	T4 (150 Days)	T5 (180 Days)
**Right Biceps Femoris**	18.9	16.4	17.0	17.1	16.8	17.9
**Left Biceps Femoris**	20.4	17.0	17.3	17.5	18.6	19.6

## Data Availability

The datasets used and/or analyzed during the current study will be made available upon reasonable request to the corresponding author, R.M.
